# Manipulation of Topological Edge States and Realization of Zero-Dimensional Higher-Order Topological Point States

**DOI:** 10.3390/mi16060686

**Published:** 2025-06-07

**Authors:** Jiahui Ren, Wenjing Ding, Sihan Wang, Shiwei Tang

**Affiliations:** School of Physical Science and Technology, Ningbo University, Ningbo 315211, China; 2211690037@nbu.edu.cn (J.R.); 2211260096@nbu.edu.cn (W.D.); 2411690049@nbu.edu.cn (S.W.)

**Keywords:** topological photonics, topological edge state, higher-order topological state

## Abstract

Topological photonics has provided revolutionary ideas for the design of next-generation photonic devices due to its unique light transmission properties. This paper proposes a honeycomb photonic crystal structure based on a mirror-symmetric interface and numerically simulates the precise manipulation of topological edge states and the robust excitation of high-order topological corner states in this structure. Specifically, two honeycomb photonic crystals with non-trivial topological properties form an interface through mirror-symmetric stitching. Continuous adjustment of the spacing between their coupling pillars can induce the closure and reopening of topological edge state energy bands, accompanied by significant band inversion, revealing the dynamic process of topological phase transitions. Furthermore, zero-dimensional high-order topological corner states are observed at the junction of boundaries with different topological properties. Their localized field strengths are strictly confined and exhibit strong robustness against structural defects. This study not only provides a new mechanism for the local symmetry manipulation of topological edge states but also lays a foundation for the design of high-order topological photonic crystals and the practical application of topological photonic devices.

## 1. Introduction

With the groundbreaking discoveries of the quantum Hall effect and topological insulators in condensed matter physics [1,2,3], the concept of topology has permeated various subfields of physics. In photonic systems, the emerging interdisciplinary field of topological photonics has rapidly become a focal point since the realization of the photonic analogue of the Haldane model in photonic crystals in 2008 [4,5]. Its central appeal lies in engineering photonic band structures with nontrivial topological invariants to achieve backscattering-immune transport channels, a capability that promises to revolutionize next-generation optoelectronic devices [6,7]. In particular, by virtue of the bulk boundary correspondence principle [8], interfaces between materials with differing topological invariants host topologically protected edge states that are robust against local perturbations.

In the early development of topological photonics, research largely adapted classification schemes from electronic systems. For instance, Chern number-based Hall-type topological phases realize unidirectional transport by breaking time reversal symmetry, albeit at the cost of requiring external magnetic fields or dynamic modulation [9]. The subsequent proposal of a quantum spin Hall analogue in photonics marked a new direction: by constructing an artificial pseudospin degree of freedom, one can obtain bidirectional topological boundary channels while preserving time reversal symmetry [10]. This advancement laid the theoretical groundwork for on-chip integrated photonic circuits. Unlike electronic systems, however, photons are bosons without intrinsic spin and thus lack a natural Kramers degeneracy. Creating pseudospin states with Kramers-like degeneracy became a central challenge. Khanikaev et al. first addressed this by exploiting double Dirac cones in a triangular lattice [10], but structural perturbations easily lifted the degeneracy. In 2015, Wu et al. introduced a honeycomb lattice unit cell in which a single triangular lattice cylinder is replaced by a six-fold symmetric array of cylinders, leveraging C6v lattice symmetry in concert with time reversal symmetry to realize robust pseudospin states [11]. Microwave experiments subsequently confirmed pseudospin-momentum locking in this design [12]. Building on these pioneering works, a variety of deformed honeycomb photonic crystal structures has been proposed, ranging from the introduction of valley degrees of freedom [13,14] to the realization of higher-order topological states [15,16].

As research has progressed, the dynamic tunability of topological edge states has emerged as a critical challenge. Traditional approaches rely on global modifications of lattice parameters, such as adjusting the size or arrangement of dielectric pillars, to open or close bandgaps and thus drive topological phase transitions [17]. These global schemes, however, lack real-time reconfigurability. Recent efforts to introduce nonlinearities [18], apply external fields [19], or employ other tuning mechanisms have achieved local control but often at the expense of response speed or symmetry integrity. In particular, tuning topological interface states typically necessitates rebuilding entire lattice sections or varying external field strengths [20,21], complicating fabrication and amplifying sensitivity to defects and process variations. Moreover, conventional trivial–nontrivial interfaces frequently break certain symmetries, preventing complete closure of the edge state bandgap and resulting in limited operational bandwidth and elevated propagation losses. Therefore, developing methods for precise, local symmetry-based control of topological edge states, without global structural reconstruction, is essential for the advancement of reconfigurable topological photonic devices. Recent theoretical proposals have suggested leveraging local defects or strain to induce tunable topological channels, effectively switching edge modes on and off, offering fresh avenues for device design [22].

Higher-order topological photonics further expands the topological paradigm by enabling localized zero-dimensional or one-dimensional modes at crystal corners or trijunctions through band inversion and multiple symmetry protections [23]. Such higher-order topological photonics provide an additional degree of freedom for topological control by exploiting specific lattice symmetries to realize dimensionally reduced modes.

In this study, we propose and experimentally validate a novel local tuning scheme based on mirror-symmetric interfaces. Two semi-infinite honeycomb photonic crystals are joined in a mirror-symmetric configuration, preserving overall time-reversal symmetry alongside a designated mirror symmetry at the interface. By finely adjusting the spacing of a single row of dielectric pillars at the interface, we achieve continuous modulation of topological edge states and observe the closing and reopening of a pair of edge state bands accompanied by clear band inversion. We demonstrate novel higher-order topological modes by exploiting the distinct edge states before and after inversion. Our findings reveal a previously unreported mechanism of band inversion for topological edge bands and furnish important theoretical insights and practical strategies for designing higher-order topological photonic crystals and tunable topological photonic devices.

## 2. Design of Mirror-Structured Topological Photonic Crystal Structures

**Figure 1:**
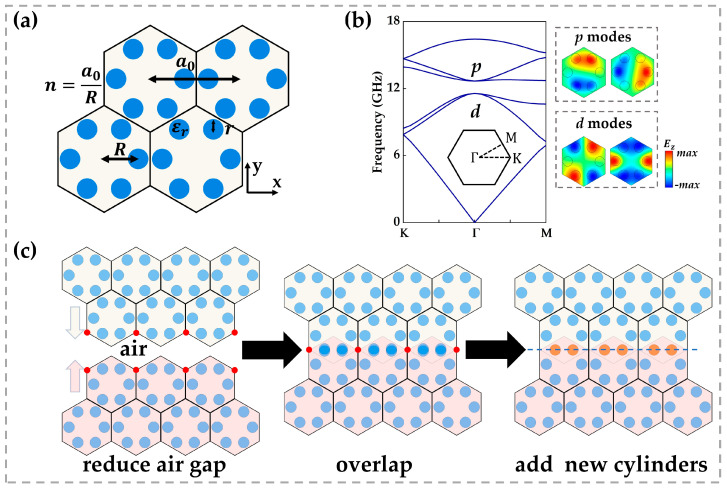
(**a**) Schematic of the honeycomb photonic crystal unit cell formed by six dielectric rods arranged with sixfold ($C_{6}$) rotational symmetry. In our simulations, the lattice constant is $a_{0}=12$ mm, each rod has a radius $r=a_{0}/9$, relative permittivity is $\varepsilon_{r}=11.7$, and it is positioned at a radial distance $R=a_{0}/2.8$ from the cell centre. (**b**) Photonic band structure of a single unit cell. The inset on the right shows the electric field profiles of the two degenerate eigenmodes at the *Γ* point. (**c**) Construction of the mirror-symmetric interface, where red markers denote lattice sites brought into coincidence by translating the two half-crystals; overlapping rods are removed and replaced by a row of coupling rods (orange); the dashed line indicates the mirror symmetry axis.

We employed a kind of honeycomb photonic crystal with sixfold rotational symmetry ($C_{6}$) as the base structure, as illustrated in Figure 1a. Six high-dielectric rods ($\varepsilon_{r}=11.7$, corresponding to silicon at microwave frequencies) were arranged in a hexagonal pattern [24]. The lattice constant was $a_{0}=12$ mm; each rod had a radius $r=a_{0}/9$ and lay at a radial distance $R=a_{0}/2.8$ from the unit-cell centre. Under these parameters, two pairs of doubly degenerate modes appeared at the Γ point, separated by complete bandgaps: the higher-frequency pair originated from odd-symmetric $p_{x}$ and $p_{y}$ orbital hybridization, while the lower-frequency pair stemmed from even-symmetric $p_{xy}$ and $p_{x^{2}-y^{2}}$ hybridization [11], as shown in Figure 1b. This band structure satisfies nontrivial topological criteria.

To reveal the formation mechanism of topological edge states, we designed a mirror-symmetric interface (Figure 1c). Two identical semi-infinite honeycomb crystals with zigzag terminations were translated toward each other along the lattice direction until lattice sites overlapped (red circles). The overlapping rods were removed, and a row of coupling rods (orange) was inserted along the same horizontal line, restoring mirror symmetry about the central axis. This configuration retains bulk topology and enables local geometric control of edge states.

**Figure 2:**
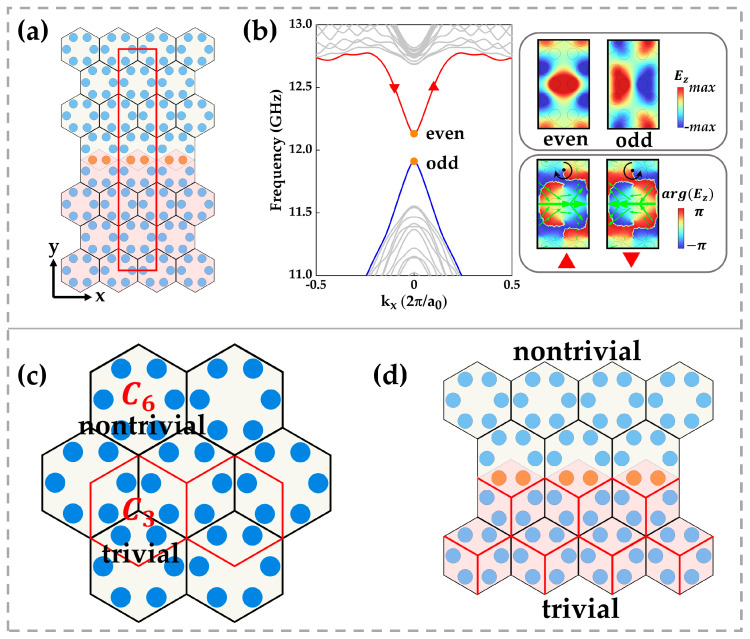
(**a**) Ribbon supercell (red rectangle) used for eigenmode calculations. (**b**) Corresponding band dispersion: solid grey curves denote bulk bands, while the solid blue and red curves indicate the edge bands. Insets on the right show the edge-state field profiles at $k_{x}=0$ (**top**) and the phase distribution of the mode marked by the red triangle (**bottom**). Black arrows indicate the direction of phase vortex winding; green arrows denote the momentum flow (time-averaged Poynting vector). (**c**) Two alternative unit cell choices in the honeycomb lattice exhibit different rotational symmetries: the cell outlined in black has $C_{6}$ symmetry, whereas the red-outlined cell has $C_{3}$ symmetry. (**d**) The mirror-symmetric interface structure can be viewed as the junction between $C_{6}$- and $C_{3}$-symmetric lattices.

We computed the eigenmodes using a ribbon supercell method (Figure 2a), in which a red rectangular supercell repeats along x to make $k_{x}$ a good quantum number, and multi-layer perfectly matched layers (PMLs) along y absorb outgoing radiation, reducing finite-size effects. All bands are calculated via the finite-difference time domain (FDTD) method, with x and y axes labelled in the supercell diagram.

Figure 2b shows the resulting dispersion: grey curves are bulk bands, and a pair of isolated topological edge states (red and blue) lies within the bandgap. These edge modes are analogous to those in a quantum spin Hall insulator with broken time-reversal symmetry, where a small gap opens when that symmetry is disrupted [25]. For $k_{x}\neq 0$, edge states carry finite orbital angular momentum (OAM), appearing as phase vortices and circulating Poynting flows in the field profiles [26,27] (inset, bottom right). At $k_{x}=0$, the OAM cancels under time reversal, and the edge-state fields restore mirror symmetry (inset, top right), with the even mode at a higher frequency than the odd mode.

In Figure 2c, we note that the same honeycomb lattice manifests two rotational symmetries: one unit cell (black outline) has $C_{6}$ symmetry, while another (red outline) shows $C_{3}$ symmetry. We use symmetry indicators at high-symmetry points Γ, M, and K to characterize band topology. For a $C_{6}$-symmetric crystal, the topological index is defined as the difference in rotation eigenvalue counts between Π (M or K) and Γ below the gap [28,29]. The resulting index χ comprises two numbers, $\left[M_{1}^{\left(2\right)}],[K_{1}^{\left(3\right)}\right]$, which for our $C_{6}$ unit cell evaluates to (2, 0), indicating a nontrivial phase. In contrast, the $C_{3}$ cell yields trivial indicators (0, 0).

By bulk boundary correspondence, an interface between χ≠0 and χ=0 phases hosts localized topological edge states. As shown in Figure 2d, juxtaposing the $C_{6}$ and $C_{3}$ crystals, where the orange rods are slightly displaced, creates a symmetrical discontinuity band that supports the observed edge modes.

## 3. Edge-State Band Inversion

**Figure 3:**
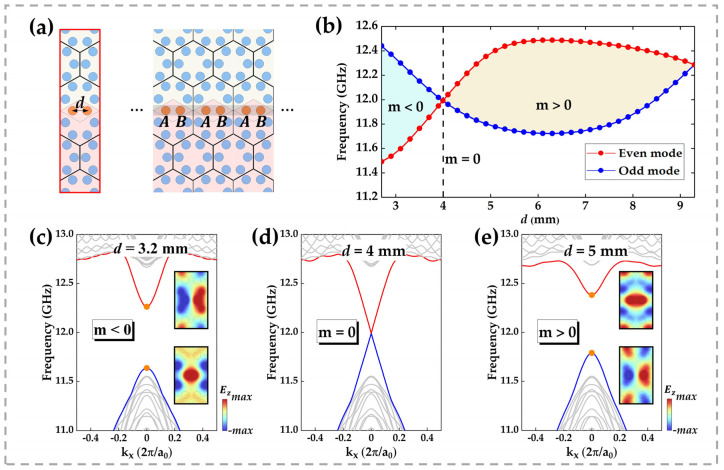
(**a**) Ribbon supercell used for eigenmode calculations, with spacing d between the two orange coupling rods. The illustration on the right shows that the two orange dielectric columns in a supercell have different statuses when coupling occurs. (**b**) Eigenfrequencies of the two modes at the Γ point as a function of rod spacing d: the blue curve denotes the odd-symmetric mode (m<0 region shaded blue), the red curve the even-symmetric mode (m>0 region shaded orange), and the two curves intersect at d=4 mm. (**c**–**e**) Band dispersions of the supercell for (**c**) d=3.2 mm, (**d**) d=4 mm, and (**e**) d=5 mm. Insets show the electric field profiles of the upper and lower edge states at $k_{x}=0$.

At the heart of our design is a row of coupling rods whose spacing, denoted d, can be continuously varied, as shown in Figure 3a. By adjusting d, we observe a striking topological phenomenon: as d increases, the two edge-state bands undergo a closing and reopening sequence, as vividly illustrated in Figure 3c–e. The insets display the electric field profiles of the two edge modes at $k_{x}=0$. When d=3.2 mm, the odd-symmetric mode lies above the even-symmetric mode at $k_{x}=0$; increasing d to 4 mm brings the two modes into degeneracy at $k_{x}=0$; further increasing d to 5 mm inverts their ordering, with the odd mode below and the even mode above at $k_{x}=0$. This progression demonstrates that the edge-state bands not only reopen but also invert as d varies.

This phenomenon can be interpreted using the Su–Schrieffer–Heeger (SSH) model [30]. As shown in the right inset of Figure 3a, we label the two coupled pillars within a supercell as A and B. When the intra-cell coupling (between A and B within the same supercell) and the inter-cell coupling (between B in one supercell and A in the adjacent supercell) are staggered, an energy gap appears between the boundary state bands. However, when the two coupling strengths are equal, this energy gap closes, enabling electromagnetic waves with frequencies inside the bulk bandgap to propagate smoothly along the boundary. As the staggered coupling induces an opening of the gap, the higher-frequency boundary state band at $k_{x}=0$ shifts downward, while the lower-frequency band shifts upward. This inversion of boundary state energy bands signifies a topological phase transition. To quantify the gap between edge modes, we introduce the Dirac mass m, defined as the frequency difference between the even and odd edge modes [31,32]:

(1) $$m=\omega_{even}-\omega_{odd},$$

where $\omega_{even}$ and $\omega_{odd}$ are the frequencies of the even- and odd-symmetric edge states at $k_{x}=0$. Figure 3b plots $\omega_{even}$ and $\omega_{odd}$ as functions of d over the range 2.7 mm≤d≤9.3 mm. It is evident that m<0 for d<4 mm (blue region) and m>0 for d>4 mm (orange region) are analogous to the topological phase transition in the one-dimensional SSH model driven by variations in inter-cell and intra-cell coupling. Notably, at d=9.3 mm, the overlapping of coupling rods between adjacent supercells reintroduces degeneracy, underscoring the role of spatial constraints on topological characteristics. This geometric parameter tuning strategy offers an intuitive and practical method for exploring topological phase transitions.

## 4. Higher-Order Topological Modes

**Figure 4:**
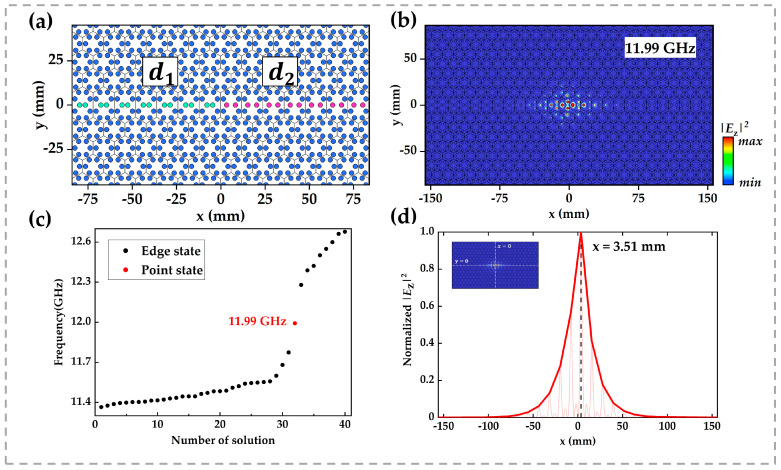
(**a**) Schematic of the composite interface between regions of distinct topology: the left section (green rods) has inter-rod spacing $d_{1}=3.2\;mm$, while the right section (magenta rods) has $d_{2}=5\;mm$. Surrounding boundaries are set to scattering conditions. (**b**) Electric field intensity distribution of the point state at f=11.99 GHz. (**c**) Eigenfrequency spectrum overlaid on the band dispersion: black dots denote edge-state eigenfrequencies, and the red dot marks the point-state frequency at 11.99 GHz. (**d**) Line profile of ${\left|E\right|}^{2}$ along y=0 through the point-state mode, illustrating field localization at the interface junction.

According to the bulk boundary correspondence, interfaces between photonic crystals with distinct topological indices host protected edge states. By extension, we hypothesize that the junctions where two different edge interfaces meet should support higher-order topological modes. To test this conjecture, we constructed a compound interface combining $d_{1}=3.2\;mm$ and $d_{2}=5\;mm$ regions, as depicted in Figure 4a, and compute its eigenfrequencies. During the simulation, we ensured that the structure was large enough and set scattering boundary conditions around it to eliminate finite size effects.

The eigenmode analysis reveals a zero-dimensional corner state within the edge-state bandgap at f=11.99 GHz, as shown in Figure 4c. Figure 4b plots the spatial distribution of its electric field intensity, and Figure 4d shows a line cut of ${\left|E\right|}^{2}$ along y=0. In both visualizations, the field is clearly localized at the interface junction, in agreement with our prediction.

**Figure 5:**
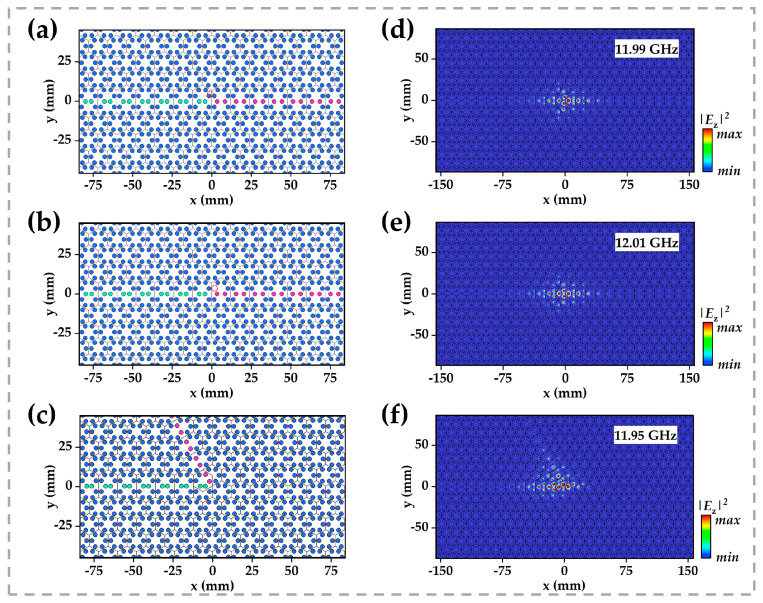
Schematic of (**a**) the photonic-crystal interface with a dielectric rod displacement defect; (**b**) the interface with a missing dielectric rod defect; (**c**) two distinct edge-state pathways negotiating a 60° corner. (**d**–**f**) Electric-field intensity distributions of the point mode at its eigenfrequency for the three configurations shown in (**a**–**c**), respectively.

To demonstrate the topological protection of this corner mode, we introduced various local perturbations, rod displacements, removals, and a 60° bend into the structure, as illustrated in Figure 5. Similarly, scattering boundary conditions were set around the structure. Although these defects induce only minor shifts in the corner-state frequency, the mode remains tightly bound to the corner, indicating robustness against structural imperfections. This stability arises from a dual-protection mechanism: the nontrivial topological invariant secures the mode’s eigenfrequency gap, while the lattice’s mirror symmetry constrains its spatial symmetry-breaking profile, yielding a symmetry-protected topological state.

Benefiting from the preserved higher-order spatial symmetries, the platform can be generalized to multi-tiered topological photonic systems. For example, by periodically arranging corner defects with differing topological charges, one can engineer synthetic dimensional photonic crystals and realize arrays of higher-order corner modes with tunable coupling and topology control.

## 5. Conclusions

In this study, through numerical simulations, we formed a mirror-symmetric interface by overlapping two identical honeycomb photonic crystals with nontrivial band topology and demonstrated a pair of interface states exhibiting spin-momentum locking. The unique properties of these topological edge modes were elucidated via the interface between $C_{6}$ and $C_{3}$ lattice symmetries. Our simulations revealed that precise geometric tuning of the coupling rod spacing induces closing and reopening and consequent inversion of the edge-state bands. At the junction of one-dimensional boundaries with distinct topological indices, we predicted and observed a novel higher-order zero-dimensional corner state. This phenomenon not only uncovers a new mechanism for band inversion in edge bands but also provides essential insight for exploring and designing higher-order topological photonic crystals.

Additionally, our local tuning scheme based on a mirror-symmetric interface, owing to its simple design and fabrication, exhibits significant potential for tunable optical devices. This approach opens new routes to achieve efficient and robust topologically protected light transport, bridging fundamental theory and practical application in topological photonics. Future work will focus on leveraging this minimalist platform to realize more complex topological photonic devices and exploring their concrete uses in information processing and communication technologies. Our findings underscore the critical role of understanding and controlling topological phase transitions and offer theoretical guidance and technological strategies to advance higher-order topological photonics.

## Data Availability

The original contributions presented in this study are included in this article; further inquiries can be directed to the corresponding authors.

## References

[1] Dorda G., Pepper M., Klitzing K.V. (1980). New method for high-accuracy determination of the fine-structure constant based on quantized Hall resistance. Phys. Rev. Lett..

[2] Kane C.L., Hasan M.Z. (2010). Colloquium: Topological insulators. Rev. Mod. Phys..

[3] Zhang S., Qi X. (2011). Topological insulators and superconductors. Rev. Mod. Phys..

[4] Chong Y., Joannopoulos J.D., Soljačić M., Wang Z. (2008). Reflection-free one-way edge modes in a gyromagnetic photonic crystal. Phys. Rev. Lett..

[5] Wang Z., Chong Y., Joannopoulos J.D., Soljačić M. (2009). Observation of unidirectional backscattering-immune topological electromagnetic states. Nature.

[6] Lu L., Joannopoulos J.D., Soljačić M. (2014). Topological photonics. Nat. Photonics.

[7] Price H.M., Amo A., Goldman N., Hafezi M., Lu L., Rechtsman M.C., Schuster D., Simon J., Zilberberg O., Carusotto I. (2019). Topological photonics. Rev. Mod. Phys..

[8] Hatsugai Y. (1993). Chern number and edge states in the integer quantum Hall effect. Phys. Rev. Lett..

[9] Lin Z., Chan C., Ao X. (2009). One-way edge mode in a magneto-optical honeycomb photonic crystal. Phys. Rev. B.

[10] Khanikaev A.B., Hossein Mousavi S., Tse W., Kargarian M., Macdonald A.H., Shvets G. (2013). Photonic topological insulators. Nat. Mater..

[11] Hu X., Wu L. (2015). Scheme for achieving a topological photonic crystal by using dielectric material. Phys. Rev. Lett..

[12] Xu Y., Xu T., Wang H., Jiang J., Hu X., Hang Z., Yang Y. (2018). Visualization of a unidirectional electromagnetic waveguide using topological photonic crystals made of dielectric materials. Phys. Rev. Lett..

[13] Yang Y., Jiang H., Hang Z. (2018). Topological valley transport in two-dimensional honeycomb photonic crystals. Sci. Rep..

[14] Huang S., Chen K.P., Rechtsman M.C., Noh J. (2018). Observation of photonic topological valley Hall edge states. Phys. Rev. Lett..

[15] Shao S., Liang L., Hu J., Poo Y., Wang H. (2023). Topological edge and corner states in honeycomb-kagome photonic crystals. Opt. Express.

[16] Yang Y., Jia Z., Wu Y., Xiao R., Hang Z., Jiang H., Xie X. (2020). Gapped topological kink states and topological corner states in honeycomb lattice. Sci. Bull..

[17] Wang H., Liang L., Jiang B., Hu J., Lu X., Jiang J. (2021). Higher-order topological phases in tunable *C*_3_ symmetric photonic crystals. Photonics Res..

[18] Maczewsky L.J., Heinrich M., Kremer M., Ivanov S.K., Ehrhardt M., Martinez F., Kartashov Y.V., Konotop V.V., Torner L., Bauer D. (2020). Nonlinearity-induced photonic topological insulator. Science.

[19] Guo K., Xue Q., Chen F., Zhou K., Liu S., Guo Z. (2021). Tunable topological valley Hall edge state based on large optical Kerr effect. J. Appl. Phys..

[20] Zhao Y., Liang F., Han J., Wang X., Zhao D., Wang B. (2022). Tunable topological edge and corner states in an all-dielectric photonic crystal. Opt. Express.

[21] Sui F., Chen J., Huang H. (2022). Tunable topological edge states and rainbow trapping in two dimensional magnetoelastic phononic crystal plates based on an external magnetostatic field. Int. J. Mech. Sci..

[22] Xu Y., Ding F., Liu F., Tang S. (2023). Continuously tunable topological defects and topological edge states in dielectric photonic crystals. Phys. Rev. B.

[23] Deng W., Shi F., Zhao F., Chen M., Dong J., Chen X. (2019). Direct observation of corner states in second-order topological photonic crystal slabs. Phys. Rev. Lett..

[24] Yang X., Liu X., Yu S., Gan L., Zhou J., Zeng Y. (2019). Permittivity of undoped silicon in the millimeter wave range. Electronics.

[25] He L., Shen Q., Xu J., You Y., Yu T., Shen L., Deng X. (2018). One-way edge modes in a photonic crystal of semiconductor at terahertz frequencies. Sci. Rep..

[26] Zhou R., Lin H., Wu Y., Li Z., Yu Z., Liu Y., Xu D. (2022). Higher-order valley vortices enabled by synchronized rotation in a photonic crystal. Photonics Res..

[27] Deng W., Chen X., Zhao F., Dong J. (2018). Transverse angular momentum in topological photonic crystals. J. Opt..

[28] Li T., Hughes T.L., Benalcazar W.A. (2019). Quantization of fractional corner charge in *C_n_*-symmetric higher-order topological crystalline insulators. Phys. Rev. B.

[29] Ghorashi A., Christensen T., Rechtsman M.C., Benalcazar W.A., Vaidya S. (2023). Topological phases of photonic crystals under crystalline symmetries. Phys. Rev. B.

[30] Asbóth J.K., Oroszlány L., Pályi A. (2016). A Short Course on Topological Insulators.

[31] Zhang X., Wang H., Lin Z., Tian Y., Xie B., Lu M., Chen Y., Jiang J. (2019). Second-order topology and multidimensional topological transitions in sonic crystals. Nat. Phys..

[32] Lin Z., Wang H., Zhang X., Lu M., Chen Y., Jiang J., Xiong Z. (2020). Corner states and topological transitions in two-dimensional higher-order topological sonic crystals with inversion symmetry. Phys. Rev. B.

